# The Role of DNA Methylation in the Metabolic Memory Phenomenon Associated With the Continued Progression of Diabetic Retinopathy

**DOI:** 10.1167/iovs.16-19759

**Published:** 2016-10

**Authors:** Manish Mishra, Renu A. Kowluru

**Affiliations:** Kresge Eye Institute, Wayne State University, Detroit, Michigan, United States

**Keywords:** diabetic retinopathy, DNA methylation, metabolic memory

## Abstract

**Purpose:**

Clinical and experimental studies have shown that diabetic retinopathy progression does not halt after termination of hyperglycemia, suggesting a “metabolic memory” phenomenon. DNA is highly dynamic, and cytosine methylation changes can last for several years. In diabetes, DNA methylation regulates expression of many genes associated with retinal mitochondrial homeostasis. Our aim was to investigate the role of DNA methylation in the metabolic memory.

**Methods:**

Reversal of 4 days of 20 mM glucose by 4 to 8 days of 5 mM glucose, in the presence/absence of Dnmt inhibitor (5-aza-2′-deoxycytidine), was investigated on DNA methylation and its machinery in human retinal endothelial cells. The key parameters were confirmed in the retina from diabetic rats maintained in good glycemic control (glycated hemoglobin ∼6%) for 3 months after 3 months of poor control (glycated hemoglobin >10%).

**Results:**

DNA methyltransferase 1 (Dnmt 1) remained active after 4 days of normal glucose that followed 4 days of high glucose, and mtDNA stayed hypermethylated with impaired transcription. Hydroxymethylating enzyme Tet2, and matrix metalloproteinase-9 (regulated by hydroxymethylation) also remained upregulated. But, 8 days of normal glucose after 4 days of high glucose ameliorated mtDNA methylation and *MMP-9* hydroxymethylation. Direct Dnmt targeting by Aza during the reversal period benefited methylation status of mtDNA and *MMP-9* DNA. Similarly, reinstitution of good control after 3 months of poor control in rats did not reverse diabetes-induced increase in retinal *Dnmt1* and *Tet2*, and alter the methylation status of mtDNA and *MMP-9*.

**Conclusions:**

Retinal DNA methylation-hydroxymethylation machinery does not benefit immediately from reversal of hyperglycemia. Maintenance of good glycemic control for longer duration, and/or direct targeting DNA methylation ameliorates continuous mitochondrial damage, and could retard/halt diabetic retinopathy progression.

Retinopathy continues to be perceived as one of the most feared microvascular complications of diabetes, and hyperglycemia remains as the major instigator of its development.^1^ The ongoing Epidemiology of Diabetes Interventions and Complications (EDIC) trial, which has followed the Diabetic Complications and Control Trial (DCCT), has clearly documented that diabetic retinopathy continues to develop and progress in individuals who were in conventional control during DCCT, but have been maintaining tight glycemic control during the EDIC phase.^2,3^ These trials have clearly suggested a “legacy” or a “metabolic memory” phenomenon, and this memory phenomenon is now well documented in experimental models and also in in vitro models of diabetic retinopathy.^4–7^ A number of metabolic and molecular events that are implicated in the development of diabetic retinopathy continue to progress even after hyperglycemic insult is replaced by a normal glycemic phase.^8–14^

Epigenetic modifications play an important role in regulation of many molecular mechanisms associated with the development of diabetic retinopathy; diabetes induces histone modifications in nuclear DNA (nDNA)-encoded genes, including matrix metalloproteinase-9 (*MMP-9*)^15^ and manganese superoxide dismutase (*Sod2*),^7^ contributing to increased oxidative stress in the retina. Hypermethylation of nDNA-encoded polymerase gamma 1 (*POLG*), important in mitochondrial DNA (mtDNA) replication, compromises mtDNA biogenesis, and that of mtDNA itself, impairs its transcription, and dysfunctions the electron transport chain system, which keeps on fueling into the vicious cycle of free radicals.^16^ Furthermore, we have shown that reinstitution of normal glycemia after a period of hyperglycemia does not reverse these epigenetic modifications, and mitochondria continue to be dysfunctional.^16,17^

DNA methylation is regulated by the opposing actions of enzymes adding methyl groups, DNA methyltransferase (Dnmt), and enzymes removing methyl groups, including ten-eleven translocase (Tet).^18,19^ Our previous work has shown that the activity of Dnmt is increased in the retina in diabetes,^20^ and among the three major members of the Dnmt family, Dnmt1, the maintenance enzyme, is upregulated.^21^ DNA methylation is a very dynamic process, and loss of methylation can occur either passively, via replication in the absence of functional maintenance methylation pathways, or actively by removing methylated cytosine by Tet, which can oxidize the methyl group of 5-methylcytosines (5mC) into 5-hydroxymethylcytosine (5hmC).^19^ Our recent work has shown that Tet2 is also activated in diabetes, and its binding at the *MMP-9* promoter is increased, allowing the promoter to be hypomethylated.^22^ However, the role of DNA methylation machinery in the metabolic memory associated with the continued progression of diabetic retinopathy remains to be investigated.

DNA is “highly dynamic” and responds to the environmental stimuli by modifying its properties in adapting to the changes,^23^ and DNA methylation and the changes brought by this can last for several years.^24^ Our hypothesis is that DNA methylation machinery continues to function aberrantly even after the hyperglycemic insult is terminated, and here we aim to investigate the role of DNA methylation machinery in the metabolic memory phenomenon. Using human retinal endothelial cells, one of the targets of the histopathology associated with diabetic retinopathy, the effect of reversal of high glucose insult on DNA methylation machinery was investigated. The effect of direct inhibition of Dnmt during the reversal of glucose insult on continued DNA methylation and its functional consequence was evaluated by supplementing a Dnmt inhibitor during the reversal phase. The key parameters were confirmed in the retina from streptozotocin-induced diabetic rats, maintained in good glycemic control after a period of poor glycemic control.

## Methods

Retinal endothelial cells were prepared from human retina (HRECs) following the methods described by Chen and associates.^25^ Cells were cultured in Dulbecco's modified Eagle medium (DMEM)-F12 (HyClone, Waltham, MA, USA) containing 10% heat-inactivated fetal bovine serum (Sigma-Aldrich Corp., St. Louis, MO, USA), heparin (50 μg/mL, Sigma-Aldrich Corp.), endothelial cell growth supplement (15 μg/mL; BD Bioscience, San Jose, CA, USA), insulin transferrin selenium (1%; Sigma-Aldrich Corp.), Glutamax (1%; Gibco-ThermoFisher Scientific, Waltham, MA, USA), and antibiotic/antimycotic (1%; Sigma-Aldrich Corp.) in an environment of 95% O_2_ and 5% CO_2_. Cells from the fifth to seventh passage were incubated in 20 mM (high) glucose for 4 days, followed by 5 mM (normal) glucose for 4 or 8 additional days in a DMEM-F12 containing 1% fetal bovine serum, 9% Nu-serum (Corning, Corning, NY, USA), 50 μg/mL heparin, 5.0 μg/mL endothelial cell growth supplement, and 1% insulin transferrin. Parallel controls included HRECs incubated in continuous 5 mM glucose or 20 mM glucose, or 20 mM mannitol (osmotic control) for the entire duration of the experiment. The cells received fresh media every 48 hours. At the end of the initial 20 mM glucose incubation period for the cells in 4d-4d and 4d-8d groups, the cells were rinsed with DMEM before changing them to 5 mM glucose medium.^12^

To investigate the effect of direct inhibition of Dnmt on the metabolic memory phenomenon, a group of cells was incubated in 20 mM glucose for 4 days, followed by 5 mM glucose containing a Dnmt inhibitor, Aza (5-aza-2′-deoxycytidine, 1 μM; Sigma-Aldrich Corp.) for 4 additional days. Additional controls included the cells incubated in 5 mM or 20 mM glucose media with 1 μM Aza for 4 to 8 days. Each experiment was performed in duplicate using three or more independent cell preparations.

Rats, Wistar (male, 200 g) were made diabetic by streptozotocin (55 mg/kg body weight) and were allowed to either remain in poor glycemic control (glycated hemoglobin GHb >11.0%; PC group) or in good glycemic control (GHb ∼6.0%; GC group) for 6 months. A group of rats was maintained in poor control for 3 months followed by good control for 3 additional months (PC-Rev). The age-matched normal rats were used as controls. The rats in the PC group received 1 to 2 IU insulin 4 to 5 times a week and those in GC received insulin twice daily (5–7 IU total). Rats were weighed two times a week their blood glucose was measured once every week and GHb every 2 months using a kit from Helena Laboratories (Beaumont, TX, USA); these methods are used routinely in our laboratory.^7,10,16^ Treatment of animals was performed according to the guidelines of National Institutes of Health Principals of Laboratory Animal Care and the ARVO Statement for the Use of Animals in Ophthalmic and Vision Research and was approved by the Institutional Animal Care and Use Committee of Wayne State University.

Gene expression was measured in the RNA isolated by Trizol reagent (Invitrogen, Carlsbad, CA, USA) using standard laboratory protocol.^11,17^ Complementary DNA was synthesized from 1 μg total RNA using High Capacity cDNA Reverse Transcription Kit (Applied Biosystems, Foster City, CA, USA), and transcript abundance was quantified by SYBR green–based quantitative real-time PCR (q-PCR) using gene- and species-specific primers (Table). The results were normalized to the cycle threshold (Ct) value from *β-actin* in the same sample, and the relative fold change in the expression of target genes was calculated using the delta Ct method.^11,15,20^

**Table:**
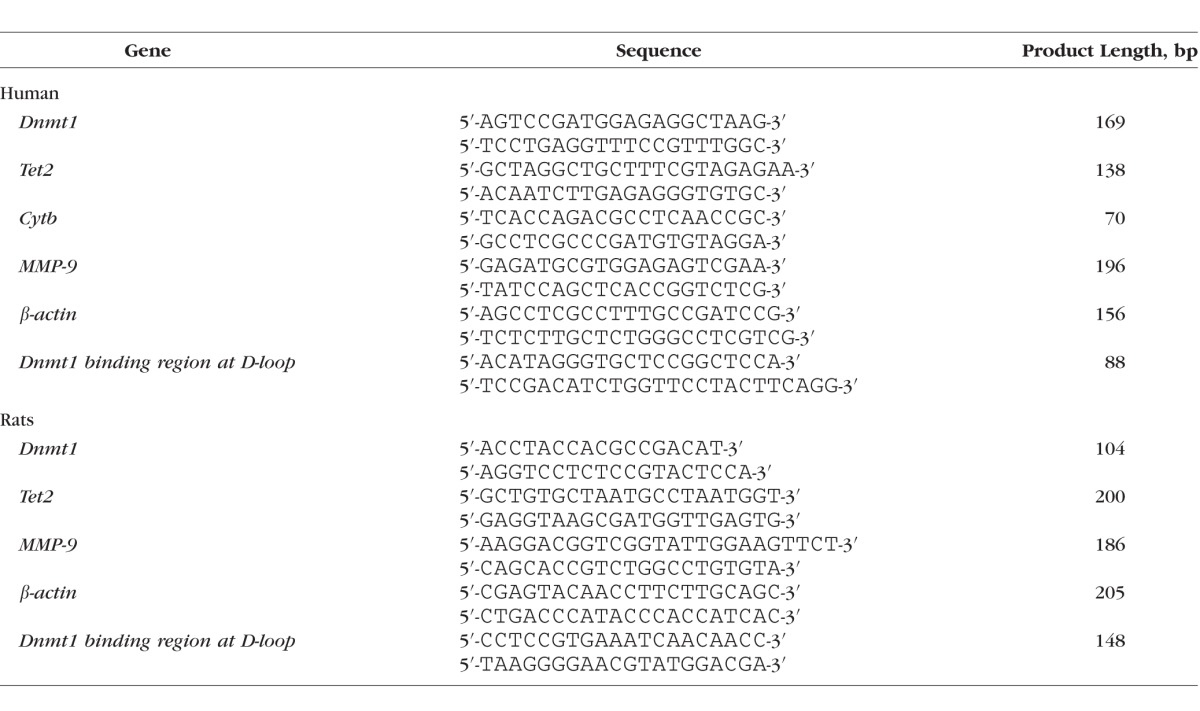
List of Primers

Immunostaining for Dnmt1 and Tet2 was performed in retinal endothelial cells incubated in normal or high glucose for 4 days using their specific antibodies (ab13537 and ab124297, respectively; Abcam, Cambridge, MA, USA), and 488 Alexa Fluor–conjugated anti-mouse (green) and Texas red–conjugated anti-rabbit (red) secondary antibodies. The coverslips were mounted using 4′,6-diamidino-2-phenylindole (DAPI) containing mounting media (blue) (Vector Laboratories, Burlingame, CA, USA), and were examined under a Zeiss ApoTome fluorescence microscope using ×40 magnification (Carl Zeiss, Inc., Chicago, IL, USA) and analyzed using ImageJ software (http://imagej.nih.gov/ij/; provided in the public domain by the National Institutes of Health, Bethesda, MD, USA).^21,26^

Quantification of 5mC was performed in the sonicated genomic DNA (100 ng), which was immunoprecipitated for 5mC by methylated DNA immunoprecipitation kit from Epigentek (P-1015–48; Epigentek, Farmingdale, NY, USA). The enriched 5mC fraction was analyzed for mtDNA (*D-loop* region) using specific primers, as reported previously.^20^

Dnmt1 binding at the *D-loop* region was assessed by Chromatin immunoprecipitation (Chip) assay. Briefly, the protein-DNA complex (120 μg) was immunoprecipitated overnight at 4°C with Dnmt1 antibody (ab13537; Abcam), and the antibody-protein-DNA complex was precipitated by protein-A agarose (Millipore, Temecula, CA, USA), washed with low salt, high salt, lithium chloride buffer, and Tris-EDTA buffer before reverse crosslinking. The samples were then digested with proteinase K, and purified DNA was analyzed by q-PCR system. Normal rabbit IgG (2729S; Cell Signaling, Cambridge, MA, USA) was used as a negative antibody control and the input DNA (40 μg protein-DNA complex) was used as an internal control. The specificity of Chip assay was confirmed by analyzing the products on a 2% agarose gel.^15,21^

Enzyme activity of MMP-9 was quantified in the homogenate (30–40 μg protein) by fluorescence kit using MMP-9–specific monoclonal antibody and a fluorogenic substrate (SensoLyte Plus 520 MMP-9 Assay Kit; AnaSpec, Inc., Fremont, CA, USA). The MMP-9–induced cleavage of the fluorogenic peptide was measured at 490-nm excitation and 520-nm emission wavelengths.^27^

### Statistical Analysis

Statistical analysis was performed with SigmaStat statistical software (Systat Software, Chicago, IL, USA) and the results are reported as mean ± SD. Data presented were analyzed statistically using the nonparametric Kruskal-Wallis test followed by Mann-Whitney *U* test for multiple group comparisons. A *P* value less than 0.05 was considered as statistically significant.

## Results

### In Vitro: Retinal Endothelial Cells

**Figure 1:**
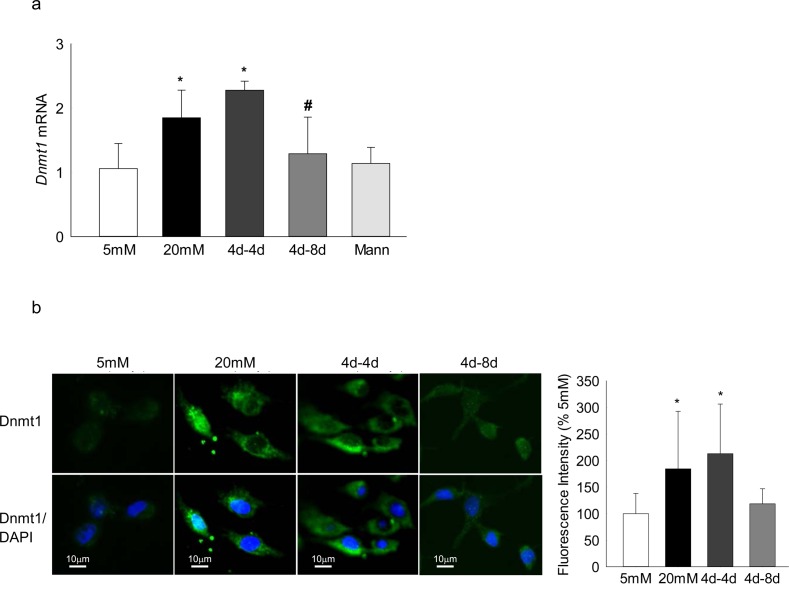
Reversal of high glucose insult on Dnmt1 in retinal endothelial cells. Retinal endothelial cells were analyzed for the expression of Dnmt1 by (**a**) SYBR green–based q-PCR using *β-actin* as a housekeeping gene, and (**b**) immunofluorescence technique using Alexa-488–conjugated secondary antibody (*green*) and DAPI (*blue*) containing mounting medium. The images are representative of three different experiments, and accompanying histogram represents mean fluorescent intensity of Dnmt1 (*green*) quantified using ImageJ software (Version 1.4.3.67); values obtained from cells incubated in continuous 5 mM glucose medium are considered as 100%. 5 mM and 20 mM = cells in 5 mM glucose or 20 mM glucose; 4d-4d and 4d-8d = 4 days of 20 mM glucose, followed by 4 and 8 days of 5 mM glucose, respectively; Mann = 20 mM mannitol. Values are mean ± SD from four to five samples per group. *P < 0.05 vs. 5 mM glucose; ^#^*P* < 0.05 vs. 20 mM glucose.

Incubation of human retinal endothelial cells in high glucose, as expected,^21^ increased *Dnmt1* expression by approximately 2-fold, and reversal of high glucose insult with normal glucose for 4 additional days (4d-4d) did not produce any beneficial effect on *Dnmt1* (Fig. 1a). Because the results from EDIC study have clearly indicated that the duration of tight control, that has followed conventional control, significantly impacts its outcome,^28^ the effect of extending duration of normal glucose to 8 days after a period of 4 days of high glucose (4d-8d) on DNA methylation was analyzed. Eight days of normal glucose exposure, after a period of 4 days of high glucose, had significant beneficial effect on *Dnmt1* gene transcripts (Fig. 1a).

To further confirm the effect of reversal of glucose insult on Dnmt1, its expression was determined by immunofluorescence technique; Figure 1b clearly shows that the levels of Dnmt1 were significantly elevated in cells incubated in continuous high glucose, and they remained elevated even when the 4 days of high glucose insult was interrupted by 4 additional days of normal glucose. However, 8 days of normal glucose after 4 days of high glucose significantly reduced expression of Dnmt1 (Fig. 1b).

**Figure 2:**
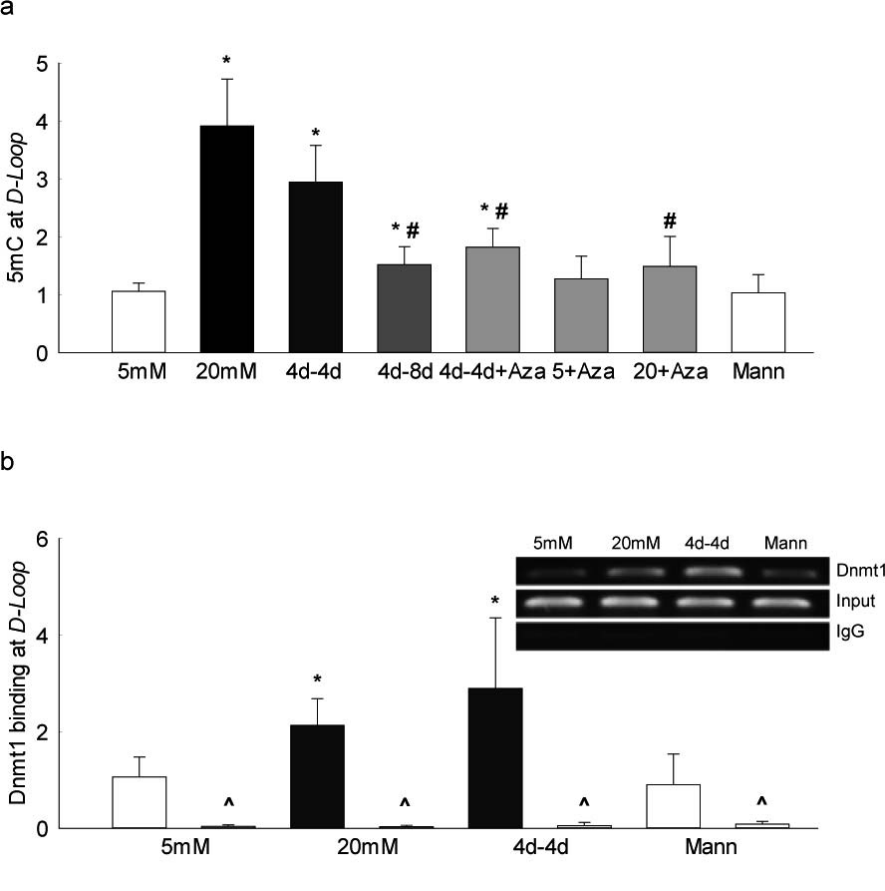
Effect of reversal of high glucose on mtDNA methylation. DNA methylation of the *D-loop* region of the mtDNA was quantified by (**a**) 5mC levels using methylated DNA immunoprecipitation technique, and (**b**) by Dnmt1 binding in the crosslinked retinal endothelial cells using Dnmt1 antibody, followed by amplification of the *D-loop* region by q-PCR; IgG was used as an antibody control (marked as ^). Fold change was calculated relative to the values obtained from cells in continuous 5 mM glucose. Data are represented as mean ± SD from each measurement made in duplicate in three to four cell preparations. 4d-4d and 4d-4d+Aza = 4 days of 20 mM glucose, followed by 4 days of 5 mM glucose in the absence or presence of Aza, respectively; 4d-8d = cells in 5 mM glucose for 4 days followed by 20 mM glucose for 8 days; 5+Aza and 20+Aza = cells in continuous 5 mM glucose or 20 mM glucose, in the presence of Aza. *P < 0.05 vs. 5 mM glucose; ^#^*P* < 0.05 vs. 20 mM glucose.

Activation of Dnmt is associated with hypermethylation, and hypermethylation of DNA results in increased levels of 5mC. In diabetes, mtDNA in retina and its endothelial cell are hypermethylated with elevated 5mC levels at its displacement loop (*D-loop*), the region of mtDNA with major transcription/replication elements.^21^ To investigate the role of DNA methylation in the metabolic memory phenomenon, 5mC levels were quantified. Continuous exposure of cells to high glucose increased 5mC levels at the *D-loop* region by 4-fold compared with cells in normal glucose, but reversal of high glucose by normal glucose for 4 additional days had no effect in normalizing 5mC levels. However, when the period of normal glucose was extended to 8 days, 5mC levels at the *D-loop* were significantly reduced compared with the values obtained from cells exposed to only 4 days of normal glucose after 4 days of high glucose, further confirming the importance of extended period of good glycemic control (Fig. 2a).

To facilitate the DNA methylation, Dnmt binds with the DNA; consistent with increased 5mC levels, Figure 2b shows that glucose-induced increase in Dnmt1 binding at the *D-loop* region also did not benefit from the reversal of glucose insult, and the values in continuous high glucose and 4d-4d groups were not different from each other, further confirming the importance of extended period of good glycemic control.

**Figure 3:**
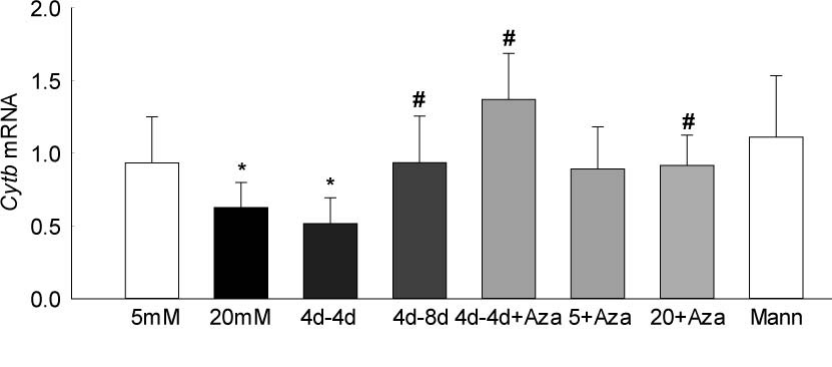
Functional consequence of continued hypermethylation on mtDNA transcription. Gene transcripts of *Cytb* were quantified by q-PCR using *β-actin* as the housekeeping gene. Relative fold changes were calculated by delta Ct method by setting the mean fraction of 5-mM glucose cells as one. Data are represented as mean ± SD from 3–4 cell preparations, with each measurement made in duplicate. *P < 0.05 vs. 5 mM glucose; ^#^*P* < 0.05 vs. 20 mM glucose.

Because hypermethylation of mtDNA compromises its transcription,^18,19^ the functional consequence of continued mtDNA hypermethylation was investigated by quantifying the gene transcripts of mtDNA-encoded Cytochrome b (*Cytb)*. As shown in Figure 3, although the transcripts of *Cytb* remain subnormal after 4 days of normal glucose, extending the normal glucose exposure to 8 days ameliorated decrease in *Cytb* transcripts; the values obtained from cells in the 4d-4d group were significantly lower compared with those from the cells in the 4d-8d group.

To determine the effect of direct inhibition of DNA methylation on metabolic memory phenomenon, the cells incubated with Dnmt inhibitor were used. Supplementation of Aza during the 4 days of normal glucose that had followed 4 days of high glucose attenuated mtDNA hypermethylation and its functional consequences, as indicated by normal levels of 5mC at *D-loop* region and *Cytb* transcripts. Levels of 5mC were significantly lower and *Cytb* transcripts were higher in the cells that were exposed to Aza during the 4 days of normal glucose that proceeded 4 days of high glucose compared with the cells in the 4d-4d group, and were similar to those obtained from the cells incubated in continuous normal or high-glucose media supplemented with Aza for the entire incubation period (Figs. 2a, 3).

**Figure 4:**
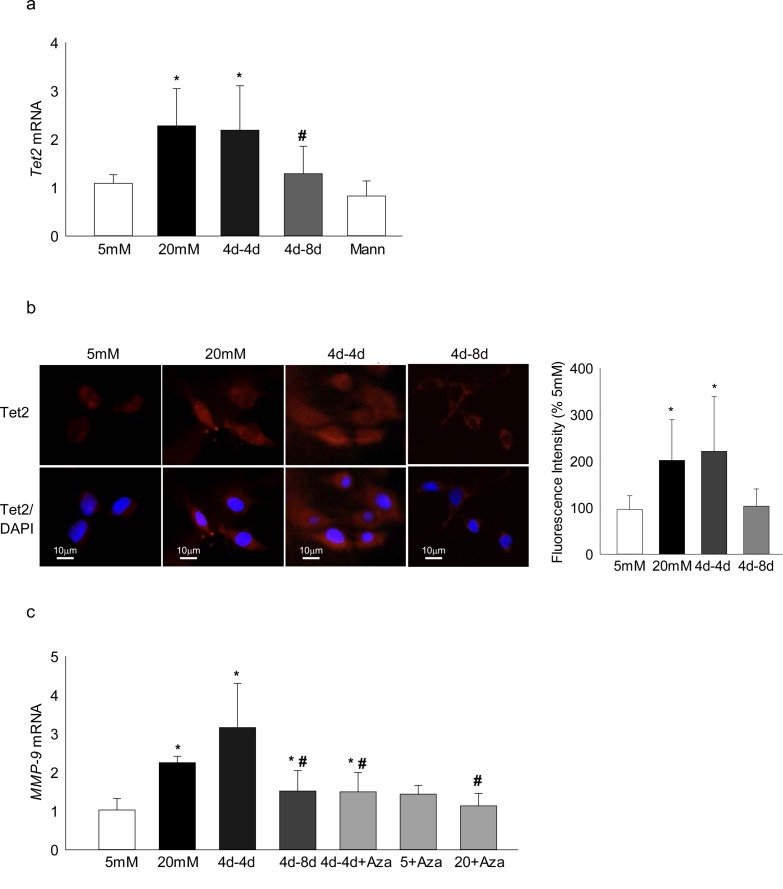
Effect of reversal of high glucose on DNA hydroxymethylation, and its functional consequence. Tet2 (**a**) gene transcripts were quantified by q-PCR using *β-actin* as the housekeeping gene, and (**b**) its expression by immunofluorescence using Texas Red–conjugated secondary antibody (*red*) and DAPI containing mounting medium (*blue*). The histogram represents mean fluorescent intensity of Tet2 (*red*) quantified using ImageJ software; values obtained from cells incubated in continuous 5-mM glucose are considered as 100%. (**c**) Gene transcripts of *MMP-9* were measured by SYBR green–based q-PCR. Data are represented as mean ± SD from each measurement made in duplicate in three to four cell preparations. *P < 0.05 vs. 5 mM glucose; ^#^*P* < 0.05 vs. 20 mM glucose.

DNA methylation is a dynamic process and methylated cytosine can be oxidized to 5hmC by Tet^23^; to understand the role of Tet in the metabolic memory phenomenon, gene transcription of *Tet2* (the only member of the Tet family upregulated in the retina in diabetes^22^), was quantified. Consistent with *Dnmt1*, *Tet2* transcripts also remained elevated even after 4 days of normal glucose that had followed 4 days of high glucose; but extending the normal glucose phase to 8 days (4d-8d), had significant beneficial effects on *Tet2* transcripts (Fig. 4a). This resistance of *Tet2* reversal was further confirmed by immunohistochemical methods, and Figure 4b clearly shows similar Tet2 expression in the cells in the continuous high-glucose and 4d-4d group; in contrast, Tet2 expression in the 4d-8d group was not different from that obtained from the cells incubated in continuous normal glucose.

Our recent work has implicated Tet2 in transcriptional activation of *MMP-9* in diabetic retinopathy^22^; to confirm the role of Tet2, the transcripts of *MMP-9* were quantified. Consistent with *Tet2*, *MMP-9* transcripts remain elevated in the cells in the 4d-4d group, but when the normal glucose phase was extended from 4 days to 8 days (4d-8d group), *MMP-9* transcripts were significantly lower compared with the values from cells in the continuous high-glucose or in 4d-4d group (Fig. 4c). Inhibition of Dnmt by supplementing Aza during 4 days of normal glucose phase, however, ameliorated increase in *MMP-9* transcripts, further confirming the role of dynamic DNA methylation-hydroxy-methylation in metabolic memory.

### In Vivo: Diabetic Rats

The role of DNA methylation in the metabolic memory associated with the continued progression of diabetic retinopathy was confirmed in a rat model. The body weights of the rats in the PC group were significantly lower and their glycated hemoglobin (GHb) levels were approximately 2-fold higher compared with the age-matched normal control rats (353 ± 41 vs. 533 ± 89 g and 11.9 ± 2.0 vs. 6.4% ± 0.6%, respectively). However, body weight and GHb values for rats in the GC group were not different from normal rats (431 ± 33 g and 6.7% ± 1.5%, respectively; *P* > 0.05). Before initiation of good glycemic control in rats that were maintained in poor glycemic control for 3 months (PC-Rev), body weight and GHb values were not different from the rats in the PC group (327 ± 47 g and 12.8% ± 0.4%, respectively), but 3 months after reinstitution of good control, the values became similar to those in the normal group (511 ± 51 g and 7.3% ± 2.1%, respectively; *P* > 0.05 versus normal).

**Figure 5:**
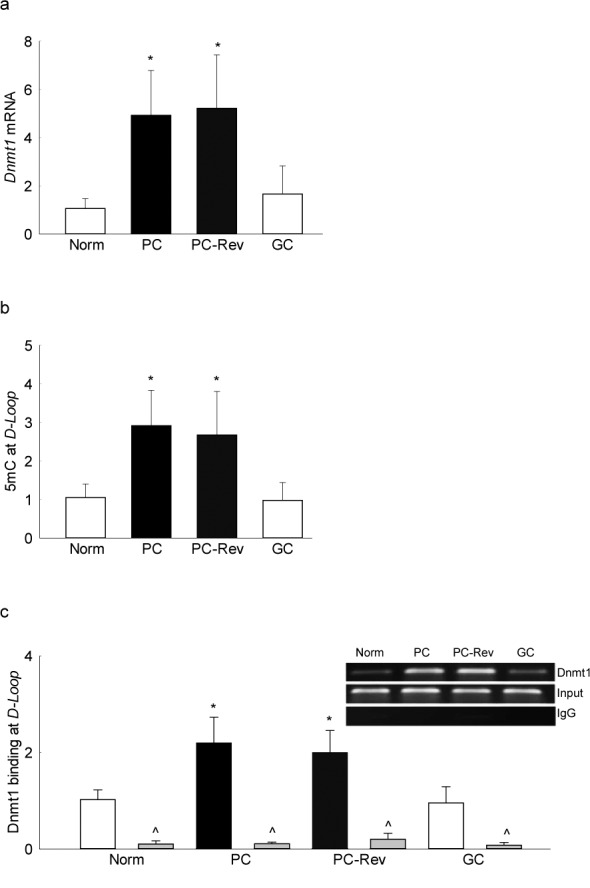
Effect of reinstitution of good glycemic control on retinal DNA methylation. Retina from rats in poor glycemic control for 3 months, followed by good control for 3 additional months was analyzed for (**a**) *Dnmt1* expression by SYBR green–based q-PCR using *β-actin* as a housekeeping gene. (**b**) Levels of 5mC at the *D-loop* region were quantified by methylated DNA immunoprecipitation technique. (c) Binding of Dnmt1 at the *D-loop* region was measured by Chip using Dnmt1 antibody, antibody control included IgG (indicated as ^). Each experiment was done in duplicate in five to six rats in each group, and the values are represented as mean ± SD. Norm, normal; Dnmt1 and IgG, crosslinked DNA immunoprecipitation with Dnmt1 antibody or IgG antibody, respectively; input, *D-Loop* abundance in total genomic DNA. *P < 0.05 compared with normal rats; #*P* < 0.05 compared with PC rats.

Reinstitution of 3 months of good control after 3 months of poor control, however, did not reverse diabetes-induced increase in retinal *Dnmt1* transcriptional activation, and the *D-loop* region remained hypermethylated with 3-fold increase in 5mC levels and 2-fold increase in Dnmt1 binding; the values were similar to those obtained from the rats in continuous poor control for the entire duration. However, reinstitution of good glycemic control immediately after induction of diabetes (GC group) protected diabetes-induced increase in *Dnmt1* transcription and mtDNA methylation in the retina, and the values obtained from the rats in the GC group were not different from the rats that remained normal (Fig. 5).

**Figure 6:**
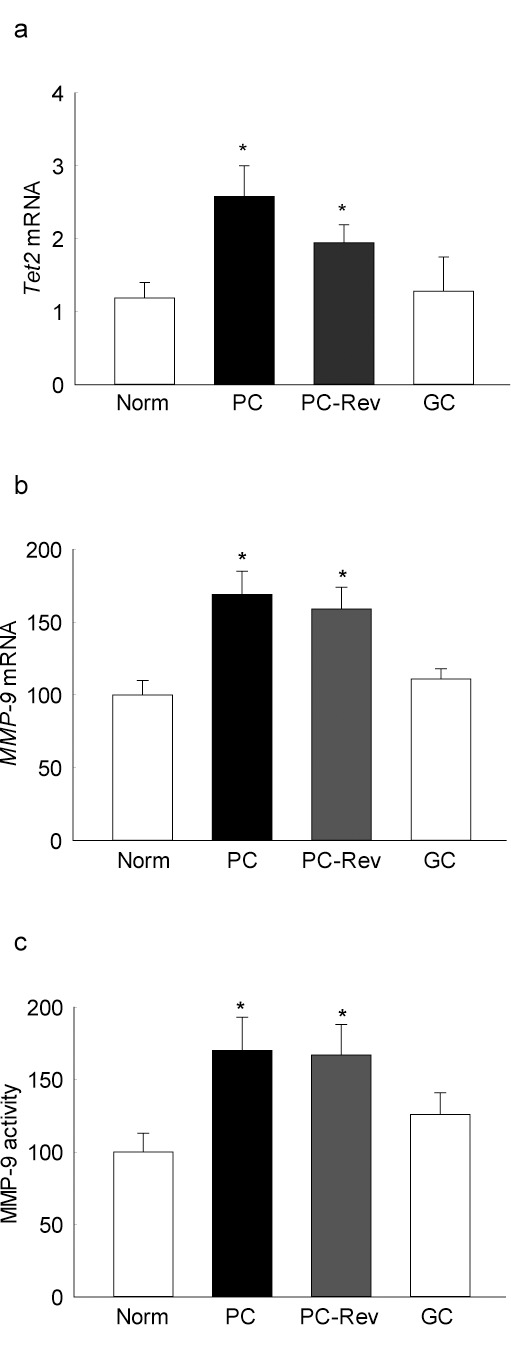
Reinstitution of good glycemic control and retinal DNA hydroxymethylation. Gene transcripts of (**a**) *Tet2* and (**b**) *MMP-9* were quantified by q-PCR using *β-actin* as the housekeeping genes. (**c**) Enzyme activity of MMP-9 was quantified fluorometrically using a 5-FAM/QXL520 FRET peptide. Data are presented as the mean ± SD from five to six rats in each group. *P < 0.05 compared with normal rats.

Consistent with Dnmt1, gene transcripts of *Tet2* in the retina also remained elevated even after 3 months of good glycemic control; *Tet2* transcripts in the rats from PC-Rev and PC groups were not different from each other (Fig. 6a). Although continuous good glycemic control prevented increase in *MMP-9* transcription, 3 months of good control that followed 3 months of poor control, however, failed to provide any beneficial effects on *MMP-9* transcription, and the values remained significantly elevated (Fig. 6b). In the same samples, MMP-9 activity continued to be upregulated even after 3 months of normal glycemia that had followed 3 months of poor glycemic control (Fig. 6c).

## Discussion

In the development of diabetic retinopathy, hyperglycemia initiates many metabolic abnormalities and induces genetic alterations in the retina and its capillary cells, and these abnormalities continue to progress even after termination of hyperglycemic insult.^4,7,9,29–31^ Mitochondria remain dysfunctional, and the futile cycle of superoxide continues to self-propagate.^10,12,17^ Recent results from EDIC have clearly documented the persistent beneficial effects of intensive therapy even 18 years after termination of DCCT.^28^ In addition to metabolic abnormalities, many epigenetic modifications are also implicated in the development of diabetic retinopathy, and these modifications by altering the conformation of DNA and its accessibility for transcriptional factors, play a significant role in regulating gene transcription.^32,33^ Experimental models have shown that the alterations in histone modifications that maintain mitochondrial homeostasis in diabetic retinopathy, including histone modifications at the glutamate-cysteine ligase, catalytic subunit-antioxidant response element 4, *Sod2* promoter/enhancer, and *MMP-9* promoter continue even after termination of hyperglycemic insult.^17,29^ Altered methylation of both mtDNA (*D-loop* region) and nDNA (*MMP-9*) are associated with mitochondrial homeostasis,^21,22^ and here we present novel results showing that reversal of hyperglycemic insult by normal glycemia does not benefit the machinery responsible for maintaining DNA methylation; enzymes responsible for DNA methylation and hydroxymethylation remain active for some time after removal of high glucose, and DNA methylation (mtDNA and nDNA) continue to be altered. The results demonstrate that the duration of normal glycemia that follows hyperglycemia (post poor glycemic control), affects the outcome of the good control; extension of this period to 8 days from 4 days has beneficial effects on DNA methylation. Furthermore, direct Dnmt targeting during the reversal phase protects continued alterations in DNA methylation machinery and ameliorates methylation of both mtDNA and nDNA. Overall, the results carry a great clinical significance, as they clearly suggest that maintaining a tight glycemic control for longer duration could eventually retard/halt the progression of diabetic retinopathy in the patients who could not, or did not, maintain a tight glycemic control during the initial stages of the disease.

DNA methylation is an important modification in long-term memory function,^33^ and the conversion of cytosine bases to 5mC is catalyzed by Dnmts.^18^ Among the Dnmt family, Dnmt1 is highly expressed in neuronal cells, and is implicated in neurodegenerative diseases, including Alzheimer's disease.^34^ Although Dnmt3a and 3b are de novo enzymes, Dnmt1 has a critical role in maintaining tissue-specific patterns of methylated cytosine.^35,36^ In diabetes, Dnmt enzyme activity is increased in the retina and its capillary cells^20^; and the expression of Dnmt1 (protein and gene), but not of Dnmt3a or 3b, is also significantly elevated.^21^ Here, our results show that Dnmt1 continues to be overexpressed even after termination of hyperglycemia, suggesting its role in the metabolic memory phenomenon. Consistent with our results, differential DNA methylation patterns are observed in the blood samples of patients with proliferative diabetic retinopathy,^37^ DNA methylation of *POLG* resists arrest.^16^ Furthermore, epigenomic profiling of the cells from subsets of DCCT/EDIC participants have clearly documented a significant role of epigenetics in the metabolic memory associated with further progression of complications during EDIC,^38^ and recent study has shown that differences in DNA methylation during the DCCT persist at certain loci associated with glycemia for several years during the EDIC study, further supporting the role of epigenetics in the metabolic memory phenomenon.^39^ However, a transient “reverse memory” effect, with worsening of retinopathy, during initial stages of tight glycemic control is also observed at times in some diabetic patients. The mechanism responsible for this “reverse memory” is unclear, and the role of epigenetic modifications in such phenomenon remains to be explored.

Mitochondria are considered to play an important role in the development of diabetic retinopathy, and their homeostasis is disturbed, and they remain dysfunctional even after termination of hyperglycemic insult.^7,10,12,16^ Dnmt1 has a mitochondrial targeting sequence, which helps its transport inside the mitochondria,^40^ and in diabetes, Dnmt1 is increased inside the retinal mitochondria and mtDNA is hypermethylated.^20^ Here, we show that reversal of hyperglycemic insult does not benefit hypermethylation of mtDNA; the binding of Dnmt1 and 5mC levels at the regulatory region of mtDNA, the region that is more prone to damage, remains elevated for a period of good glucose exposure. Because hypermethylation is associated with transcription inactivation^16,41–43^; consistent with mtDNA hypermethylation, in the same samples the gene transcripts of mtDNA-encoded proteins also remain subnormal, further fueling into the vicious cycle of free radicals. This failure to ameliorate hypermethylation, which was initiated by the prior glycemic control, suggests that mtDNA hypermethylation could be playing an important role in continued imbalance in mitochondrial homeostasis that the retina experiences during its resistance to reverse retinopathy after reinstitution of good glycemic control.

DNA methylation is a dynamic process, while Dnmt inserts a methyl group, Tet demethylates DNA by oxidizing that methyl group to 5hmC.^18,19^ Recent studies have clearly indicated that 5hmC can act not only as an intermediate in the DNA demethylation, it also serves as an independent epigenetic marker in regulating gene expression, and both active DNA methylation and demethylation are now considered as crucial regulators of gene transcription.^19^ We have shown that diabetes increases Tet activity in the retina and its capillary cells, and increased Tet2 plays a significant role in the upregulation of *MMP-9*, an enzyme implicated in mitochondrial damage. Due to increased binding of Tet2 at the *MMP-9* promoter, the levels of 5hmC are increased and hypomethylation of the promoter results in its transcriptional activation.^22^ Novel results presented here show that once the retinal Tet is activated in diabetes, it does not benefit from the good glycemic control for a duration that follows the hyperglycemia, and the *MMP-9* promoter continues to be hypomethylated with *MMP-9* activation, further compromising mitochondrial homeostasis.

Follow-up EDIC studies have shown that despite nearly similar HbA1c values at the end of DCCT, the risk of further progression of retinopathy remains significantly lower for more than 18 years for the patients who were in intensive control during DCCT; although in 4 years of EDIC follow-up, the risk reduction was 71% in the intensive group, at 18 years of follow-up, the risk reduction was decreased to 46%.^28,44^ Here we show that extension of duration of normal glucose from 4 days to 8 days, after 4 days of high glucose insult, ameliorates increase in DNA methylation and its machinery; these results further confirm the benefits of extended duration of good glycemic control, and are supported by our previous study showing that the extension of good control after a period of poor control has better effects on capillary cell apoptosis when the duration is extended.^5,9^

Reestablishment of good glycemic control, soon after induction of diabetes, however, protects retinal mitochondrial homeostasis and the progression of diabetic retinopathy.^12,16,45^ Here, we demonstrate that Dnmt and Tet also remain normal, with no change in mtDNA and nDNA (*MMP-9*) methylation. This clearly suggests that the high dose of insulin, administered to maintain good glycemic control, does not affect DNA methylation and its machinery, and strongly strengthens the need for an early and sustained good glycemic control for diabetic patients.

Regulation of Dnmt1 inhibits glucose-induced mtDNA methylation in retinal endothelial cells, and ameliorates its transcription.^21^ Here, we show that incubation of cells with an inhibitor, which inhibits DNA methylation by incorporating into DNA, when added during the reversal phase, protects glucose-induced mtDNA hypermethylation and its detrimental effects on mtDNA transcription, further strengthening the role of DNA methylation in the metabolic memory phenomenon. Moreover, supplementation with Aza also attenuates increase in *MMP-9* transcripts, suggesting that due to inhibition of DNA methylation, Tet substrate (5mC) availability is decreased, resulting in amelioration of the hypomethylation process. In support, direct regulation of increased oxidative stress during the good metabolic control, which has followed poor glycemic control, ameliorates impaired mitochondria biogenesis, and attenuates continued progression of diabetic retinopathy.^12^

We recognize that although our in vitro model used isolated retinal endothelial cells, the in vivo model used whole retina, a tissue that has complex cellular structure and multiple layers. In addition to retinal vascular cells, diabetes also damages neuronal cells and photoreceptors, and mitochondrial damage in photoreceptors is considered as one of the major contributors of retinal oxidative stress and inflammation in diabetes.^46–48^ The effect of reversal of hyperglycemia on DNA methylating machinery in other retinal cells in the metabolic memory phenomenon, however, cannot be ruled out.

In summary, our study has highlighted the importance of DNA methylation in the continued progression of diabetic retinopathy, and has provided a molecular mechanism for continued mitochondrial damage and importance of duration of good glycemic control in the metabolic memory phenomenon. Due to sustained activation of DNA methylation machinery in the retina and its capillary cells even after reversal of hyperglycemic insult, DNA methylation (nDNA and mtDNA) continues to be altered. Due to hypermethylation of DNA, the transcription of genes, including those important for mitochondrial homeostasis, remains compromised and the dysfunctional mitochondria continues to fuel into the progression of diabetic retinopathy. However, extension of the good glycemia, which has followed a period of poor glycemia, provides some benefit, and direct inhibition of Dnmt during this reversal phase helps attenuate DNA methylation and mitochondrial damage. These interesting results could have remarkable translational significance, as they show the benefits of adding targeting therapy during the good glycemic phase, and also strengthens the need for educating patients about the long durations they might take before they could see any benefits of maintaining good glycemic control.
